# Clinical characteristics, drug resistance profiles, and inflammatory cytokine signatures in newly treated versus retreated MDR/RR-TB patients: associations with treatment outcomes

**DOI:** 10.3389/fmicb.2026.1871422

**Published:** 2026-07-01

**Authors:** Yiyan Song, Dawei Yu, Junchi Xu, Yunxia Zhai, Huafeng Song, Hui Chen, Jianping Zhang, Ping Xu, Fanghua Li

**Affiliations:** 1 Department of Clinical Laboratory, The Fifth People’s Hospital of Suzhou, The Affiliated Infectious Diseases Hospital of Soochow University, Suzhou, China; 2 Department of Tuberculosis, The Fifth People’s Hospital of Suzhou, The Affiliated Infectious Diseases Hospital of Soochow University, Suzhou, China

**Keywords:** cross-resistance, inflammatory cytokines, MDR/RR-TB, MIC, treatment outcome

## Abstract

**Background:**

Multidrug-resistant/rifampicin-resistant tuberculosis (MDR/RR-TB) poses a significant threat to global tuberculosis control. Understanding drug resistance profiles, immune signatures, and their associations with treatment outcomes is critical.

**Methods:**

A total of 395 MDR/RR-TB patients were enrolled. Baseline characteristics, minimum inhibitory concentrations (MICs) for multiple anti-TB drugs, plasma inflammatory cytokine levels, and treatment outcomes were analyzed.

**Results:**

The cohort was predominantly male (69.6%), with a median age of 40 years. Pre-extensively drug-resistant (Pre-XDR-TB) accounted for 48.4%, and treatment success rate was 66.8%. Retreated patients showed significantly higher MICs for ethambutol, isoniazid, rifampicin, moxifloxacin, ofloxacin, and streptomycin (all *p* < 0.05). Strong cross-resistance was observed between moxifloxacin and ofloxacin (*ρ* = 0.93, *p* < 0.0001). Retreated patients had lower IL-2 (*p* = 0.035) and higher IL-12 levels (*p* = 0.002). Unadjusted analyses identified significant associations between elevated MICs of isoniazid, moxifloxacin, ofloxacin, and streptomycin and treatment failure. After multivariable adjustment for age, sex, treatment history, and diabetes, ethambutol (aOR = 1.620, *p* = 0.007) and isoniazid (aOR = 1.315, *p* = 0.018) showed positive associations with treatment failure, though these did not remain significant after false discovery rate correction for multiple comparisons.

**Conclusion:**

In MDR/RR-TB patients, elevated ethambutol and isoniazid MICs were associated with treatment failure after multivariable adjustment; however, these associations were not significant after multiple comparisons correction. The potential prognostic value of quantitative MIC testing for these drugs remains to be established in future adequately powered studies.

## 1 Introduction

Multidrug−/rifampicin-resistant tuberculosis (MDR/RR-TB) remains a major challenge to global tuberculosis (TB) control. According to Global tuberculosis report 2024 from World Health Organization (WHO), 175,923 people were diagnosed and treated for MDR/RR-TB globally in 2023, yet treatment success rates remain persistently low (World Health Organization, 2024). MDR/RR-TB is not a homogeneous condition. Patients differ substantially in their treatment history, which significantly influences drug resistance profiles and clinical outcomes (Soedarsono et al., 2023). Newly treated patients typically acquire resistance through primary transmission of resistant strains, whereas retreated patients often develop resistance progressively through repeated drug exposure, inadequate prior regimens, or poor adherence (Cheng et al., 2021). This distinction is clinically important, as retreated patients are more likely to harbor complex resistance patterns, including elevated minimum inhibitory concentrations (MICs) for multiple drugs and accumulation of resistance-associated mutations (Kuhlin et al., 2021).

Conventional categorical drug susceptibility testing (DST) fails to capture subtle shifts in resistance, whereas minimum inhibitory concentration (MIC) testing provides a continuous measure of bacterial susceptibility and can detect low-level resistance and stepwise MIC elevations potentially linked to prior treatment (Miotto et al., 2018; Forsman et al., 2019). Tang et al. (2022) reported that MIC values of isoniazid and fluoroquinolones were lower in newly treated *vs.* retreated MDR/RR-TB patients and associated with treatment outcomes. Recent evidence suggests that even moderately elevated MICs, while still classified as susceptible by current breakpoints, may be associated with poor clinical outcomes (Lin et al., 2021). Another important aspect of MDR/RR-TB is the phenomenon of cross-resistance among different drugs (Liu Y. H. et al., 2021; Ushtanit et al., 2023). Thus, understanding these differences between newly treated and retreated MDR/RR-TB patients is essential for guiding appropriate treatment strategies, and understanding cross-resistance patterns is critical for rational regimen design.

Beyond microbiological factors, host immunity plays a crucial role in determining treatment outcomes (Rahlwes et al., 2023; Russell et al., 2025; Sayes et al., 2026). Key cytokines involved in anti-tuberculosis immunity include pro-inflammatory mediators such as tumor necrosis factor-alpha (TNF-*α*), interleukin-6 (IL-6), and interleukin-1β (IL-1β), which promote macrophage activation, granuloma formation, and bacterial clearance (Carabalí-Isajar et al., 2023; Vats et al., 2024). Th1-type cytokines, particularly interferon-gamma (IFN-*γ*) and interleukin-12 (IL-12), are critical for driving cell-mediated immunity, while regulatory cytokines such as interleukin-10 (IL-10) modulate inflammation and prevent immunopathology (Luo et al., 2021; Li et al., 2024). Interleukin-2 (IL-2) is essential for T-cell proliferation and maintenance of memory responses (Niu et al., 2022). Yu et al. (2023) demonstrated that inflammatory ratios including the C-reactive protein to albumin ratio (CAR), neutrophil to lymphocyte ratio (NLR), and monocyte to lymphocyte ratio (MLR) predicted mortality in RR/MDR-TB patients. However, direct measurement of individual cytokines in newly treated *vs.* retreated patients remains unexplored.

In this study, we aimed to: (1) characterize the baseline clinical features, drug resistance patterns, and treatment outcomes in a cohort of 395 MDR/RR-TB patients; (2) compare MICs of multiple anti-TB drugs between newly treated and retreated patients; (3) evaluate cross-resistance among key drug pairs; (4) assess plasma levels of inflammatory cytokines and compare them between the two groups; and (5) analyze the association between MIC ranges and treatment outcomes. This study extends prior work by simultaneously evaluating MIC-based resistance profiles and individual cytokine levels in the same cohort, providing an integrated microbiological-immunological comparison between newly treated and retreated MDR/RR-TB patients.

## 2 Materials and methods

### 2.1 Study setting and participants

This retrospective study enrolled patients diagnosed with multidrug-resistant or rifampicin-resistant tuberculosis (MDR/RR-TB) at the Fifth People’s Hospital of Suzhou (Infectious Disease Hospital Affiliated to Soochow University) between December 1, 2022 and November 30, 2025. Inclusion criteria were: (1) confirmed diagnosis of MDR-TB or RR-TB; (2) HIV-negative status; and (3) diagnosis verified by mycobacterial culture, MIC drug susceptibility testing (DST), and Xpert MTB/RIF, performed according to WHO guidelines. If DST results conflicted with clinical findings, DST was repeated using another respiratory sample. Exclusion criteria were: (1) HIV infection; (2) non-tuberculous mycobacteria (NTM) infection. All patients performed MIC DST before treatment, followed by mycobacterial culture every 3 months throughout the treatment course.

### 2.2 Definitions of RR, MDR, pre-XDR, and XDR-TB

According to modern WHO guidelines, MDR-TB refers to patients with an MTB culture DST or Xpert MTB/RIF result showing resistance to at least isoniazid and rifampicin. Pre-XDR-TB refers to patients with an MTB culture DST or Xpert MTB/RIF result showing resistance to rifampicin, plus resistance to fluoroquinolones. Extensively drug-resistant TB (XDR-TB) refers to patients with the result showing resistance to rifampicin, plus resistance to fluoroquinolones and at least one Group A drug, specifically bedaquiline or linezolid. Because this study lacked bedaquiline or linezolid DST, XDR-TB cannot de confidently classified. RR-TB refers to patients with an MTB culture DST or Xpert MTB/RIF result showing resistance to rifampicin; of note, RR-TB encompasses both MDR-TB and pre-XDR-TB.

### 2.3 Treatment history and outcome classification

Newly diagnosed MDR/RR-TB refers to patients with no prior anti-TB treatment history or prior treatment less than 1 month. Re-treated MDR/RR-TB refers to patients with prior anti-TB treatment history for more than 1 month.

Treatment outcomes for MDR-TB were evaluated according to WHO guidelines. The treatment outcomes were classified into “cure,” “treatment completed,” “failure,” “death,” “lost to follow-up” and “not evaluated.” The “cure” is patients who complete the treatment with consistently at least three negative culture results for the final 12 months of the treatment course and no evidence of failure. “Treatment completed” was determined by bacterial negative conversion at the treatment end but with fewer than three negative cultures. “Cure” and “treatment completed” groups were combined into the “success” group. The “failure” was patients who had sputum culture-positive in the final 12 months of treatment, or any positive result among the last three cultures, or premature discontinuation due to clinical/radiological adverse reactions or events. “Death” was for patients who died during anti-TB treatment for any reason. “Lost to follow-up” was patients whose TB treatment were interrupted for at least two consecutive months for any reason. “Not evaluated” is patients with no treatment outcome, whose treatment period is less than 12 months and who are still undergoing treatment.

### 2.4 MTB culture and identification

Clinical specimens were cultured using the BACTEC MGIT 960 system (Becton Dickinson, MD, United States) according to the manufacturer’s instructions. Positive isolates were verified by the MPB64 antigen detection (Genesis, Hangzhou, China), cases of non-tuberculous mycobacteria (NTM) were excluded.

### 2.5 Minimum inhibitory concentration (MIC) DST of the drugs

MIC DST was performed using the Myco TB system (ThermoFisher Scientific). All laboratory procedures were conducted by trained personnel in biosafety cabinets in accordance with relevant guidelines. *Mycobacterium tuberculosis* H37Rv (ATCC 27294) was used as the reference control strain. Clinical isolates of *M. tuberculosis* and the H37Rv control strain were cultured in Middlebrook 7H9 broth supplemented with oleic acid-albumin-dextrose-catalase (OADC). The inoculum was adjusted to a concentration of 1 × 10^5^ CFU/mL (5 × 10^4^–5 × 10^5^ CFU/mL). Subsequently, 100 μL of the bacterial inoculum was added to each well of the MTB MIC plate. The plate was incubated at 37 °C and growth status in the control wells was assessed after 7–10 days of incubation. If the growth of the strain was insufficient to be performed by DST after being cultured for 10 days, it was re-incubated in the plate for up to an additional 11 days. The MIC value was defined as the lowest drug concentration that inhibited bacterial growth. Critical concentrations for drug susceptibility testing were based on CLSI M24-A2 standards and FDA-approved criteria. Results were read using the Vizion® System. A strain was considered resistant to a given drug if its MIC was equal to or higher than (≥) the respective cut-off concentration. MIC DST results were reported as quantitative data in μg/mL. The recommended cut-off concentrations were as follows: amikacin, 4 μg/mL; cycloserine, 25 μg/mL; ethambutol, 5 μg/mL; isoniazid, 0.2 μg/mL; kanamycin, 5 μg/mL; aminosalicylic acid, 2 μg/mL; rifampicin, 1 μg/mL; moxifloxacin, 0.5 μg/mL; ofloxacin, 2 μg/mL; streptomycin, 2 μg/mL; ethionamide, 5 μg/mL.

### 2.6 X-pert MTB/RIF assay

*Mycobacterium tuberculosis* identification and rifampicin resistance detection were performed using the X-pert MTB/RIF assay (Cepheid, Sunnyvale, CA, United States) according to the manufacturer’s instructions. Briefly, sputum specimens were collected from enrolled patients and mixed with the sample reagent (containing NaOH and isopropanol) at a 2:1 ratio (sample reagent to sputum) and incubated for 15 min at room temperature with intermittent shaking. Following complete liquefaction, 2 mL of the processed sample was transferred into the X-pert MTB/RIF cartridge, which was then loaded onto the GeneXpert Dx System (Cepheid). The system performs fully automated nucleic acid amplification using real-time PCR to detect the presence of *M. tuberculosis* complex DNA and mutations in the *rpoB* gene (rifampicin resistance-determining region). Results were automatically generated within approximately 90 min, reporting: (1) *M. tuberculosis* detected/not detected, and (2) rifampicin resistance detected/not detected or indeterminate. Internal sample processing controls and probe check controls were used to ensure assay validity. Specimens with indeterminate results were retested once using residual sample material.

### 2.7 Detection of plasma inflammatory cytokines

Plasma levels of 12 cytokines (IL-1β, IL-2, IL-4, IL-5, IL-6, IL-8, IL-10, IL-12p70, IL-17, IFN-*γ*, TNF-*α*, and IFN-α) were measured using a multiplex microsphere-based flow cytometry immunofluorescence assay (12-item Cytokine Detection Kit, Qingdao Ruiskaier Biotechnology Co., Ltd., China). The assay was performed according to the manufacturer’s instructions. Briefly, plasma samples were collected using EDTA anticoagulant tubes and centrifuged at 1000 × *g* for 10 min. Capture antibody-coated microspheres were vortexed for 30 s and gently pipetted approximately 30 times. A 1 × wash buffer was prepared by diluting the 10 × wash buffer with deionized water. Calibrators were reconstituted and serially diluted 4-fold (from 10,000 pg/mL to 2.44 pg/mL) using assay buffer. For each standard or sample tube, 25 μL of matrix B (for standards) or assay buffer (for samples) was added, followed by 25 μL of standard or sample, 25 μL of mixed capture microspheres, and 25 μL of biotin-labeled detection antibody. The mixture was incubated for 2 h at room temperature in the dark with shaking at 400–500 rpm. Then, 25 μL of streptavidin-phycoerythrin (SA-PE) was added to each tube, followed by another 30-min incubation under the same conditions. After incubation, 1 mL of 1 × wash buffer was added, and the tubes were centrifuged at 300–500 × *g* for 5 min. The supernatant was carefully discarded, and the pellet was resuspended in 150–300 μL of 1 × wash buffer. The samples were then analyzed on an Agilent NovoCyte flow cytometer. Cytokine concentrations were calculated from the standard curve. The detection range of the assay was 2.44–10,000 pg/mL.

### 2.8 Statistical analysis

The statistical analysis was performed using SPSS 16.0 and OriginPro 2024. Baseline data were compared between the newly treated and retreated groups based on MIC values and levels of inflammatory factors. Categorical variables were presented as frequencies and percentages and were compared using the chi-square test or Fisher’s exact test. Continuous variables were expressed as medians with quartiles. For normally distributed data, the t-test was used to compare the means of continuous variables; otherwise, the Mann–Whitney *U* test was applied. Treatment outcomes, including success and failure, were compared using Chi-square analysis. Spearman correlation analysis was conducted to examine the relationships between the MIC values of similar drugs. Spearman’s rank correlation coefficients (*ρ*) were calculated to indicate the strength and direction of the associations. The absolute value of *ρ* reflects the strength of the correlation: |*ρ*| > 0.8 indicates a strong correlation, 0.5 < |*ρ*| ≤ 0.8 indicates a moderate correlation, 0.3 < |*ρ*| ≤ 0.5 indicates a weak correlation, and |*ρ*| ≤ 0.3 indicates a very weak or no correlation. The sign of *ρ* (positive or negative) indicates the direction of the relationship. To assess independent associations, multivariable logistic regression was performed with treatment failure as the outcome. MIC values were log₂-transformed and entered as continuous variables. Separate models were constructed for each of the 12 drugs, adjusting for age, sex, treatment history, and diabetes. Results are reported as adjusted odds ratios (aOR) with 95% confidence intervals (CIs). A difference was considered statistically significant when the *p* value was less than 0.05. To account for multiple comparisons across different drugs, the Benjamini-Hochberg false discovery rate (FDR) correction was applied using Microsoft Excel. An FDR-adjusted *q*-value < 0.05 was considered statistically significant.

## 3 Results and discussion

**SCHEME 1:**
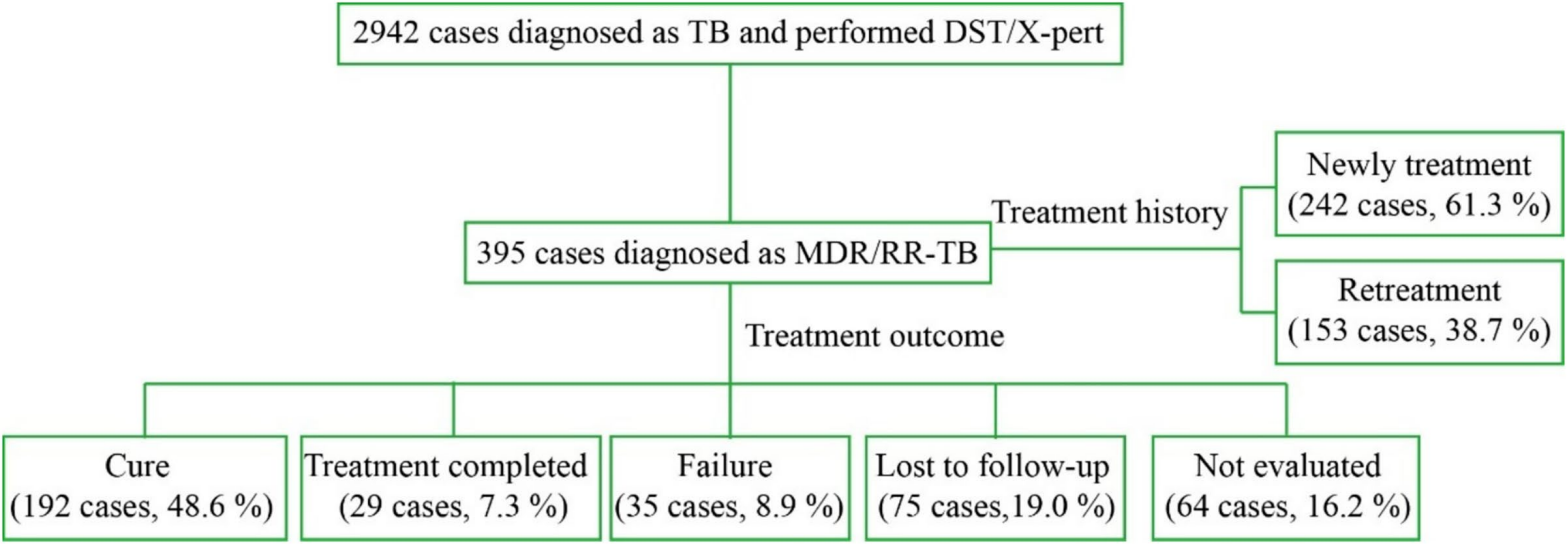
Flowchart of patient enrollment, treatment history, and clinical outcomes among MDR/RR-TB cases.

A total of 2,942 patients diagnosed with tuberculosis (TB) who underwent drug susceptibility testing (DST) or X-pert assay were initially screened. Among them, 395 patients (13.5%) were identified as having multidrug-resistant or rifampicin-resistant tuberculosis (MDR/RR-TB) and were included in the final analysis. As shown in Scheme 1, among the MDR/RR-TB patients, 242 (61.3%) were newly treated cases, while 153 (38.7%) had a history of prior treatment.

The baseline clinical characteristics of the 395 MDR/RR-TB patients are summarized in Table 1. The cohort was predominantly male (69.6%), with a median age of 40 years (interquartile range: 32–62), which is consistent with the known epidemiological profile of TB in many burden settings (Jeon et al., 2011; Akhmedullin et al., 2025). Regarding drug resistance patterns, 344 patients (87.1%) were classified as MDR-TB, and 191 (48.4%) as pre-XDR-TB. The high proportion of pre-XDR-TB is particularly concerning, as it reflects the evolving spectrum of drug resistance and poses significant challenges to standardized treatment regimens. Comorbidities were common in this cohort, and only 116 patients (29.4%) did not have any complications. Diabetes mellitus was the most frequent comorbidity, affecting 103 patients (26.1%), followed by anemia (15.9%), hypertension (11.9%), hypoproteinemia (8.9%), hepatitis (6.3%), bronchiectasis (5.6%), and chronic obstructive pulmonary disease (COPD) (4.3%). The high prevalence of diabetes is noteworthy, as it has been associated with poor TB treatment outcomes, including delayed sputum conversion and increased mortality (Choi et al., 2014; Nathella and Babu, 2017; Koo et al., 2020). With respect to extrapulmonary involvement, bronchial TB was the most frequent manifestation (26.8%), followed by pleural TB (6.8%) and tuberculous lymphadenitis (3.8%). Notably, 58.7% of patients presented with pulmonary TB only, suggesting that extrapulmonary forms are not rare and require careful diagnostic evaluation. Regarding treatment outcomes among the MDR/RR-TB patients, 192 (48.6%) achieved cure and 29 (7.3%) completed treatment, resulting in an overall treatment success rate of 66.8% [221/(395–64)]. This success rate is below the WHO target of ≥75% for MDR-TB treatment, underscoring the substantial challenges in managing this population. Treatment failure occurred in 35 patients (8.9%) and no deaths occurred, while 75 patients (19.0%) were lost to follow-up. According to WHO outcome definitions, the proportion of unfavorable events including lost to follow-up, failure, or death was 27.9%. These findings emphasize the need for comprehensive patient support strategies, including adherence counseling, side effect management, and socioeconomic assistance, to improve treatment retention and outcomes in MDR/RR-TB patients.

**Table 1:** Clinical characteristics in included patients with MDR/RR-TB.

Characteristics		Include patients
Gender	Male	275 (69.6%)
	Female	120 (30.4%)
Age		40 (32, 62)
Drug resistance type	MDR-TB	344 (87.1%)
	Pre-XDR-TB	191 (48.4%)
Treatment history	Newly treated	242 (61.3%)
	Retreated	153 (38.7%)
Treatment outcome	Cure	192 (48.6%)
	Treatment completed	29 (7.3%)
	Success	221 (55.9%)
	Failure	35 (8.9%)
	Death	0 (0.0%)
	Lost to follow-up	75 (19.0%)
	Not evaluated	64 (16.2%)
Complications	Anemia	63 (15.9%)
	Hepatitis	25 (6.3%)
	Diabetes mellitus	103 (26.1%)
	Hypertension	47 (11.9%)
	COPD	17 (4.3%)
	Hypoproteinemia	35 (8.9%)
	Bronchiectasis	22 (5.6%)
	Cardiopathy	5 (1.3%)
	None	116 (29.4%)
With extra-pulmonary TB	Bronchial TB	106 (26.8%)
	tuberculosedadenitis	15 (3.8%)
	Pleural TB	27 (6.8%)
	Bone TB	4 (1.0%)
	Cutaneou TB	2 (0.5%)
	Meningeal TB	5 (1.3%)
	Peritoneal TB	2 (0.5%)
	Urinary system TB	1 (0.3%)
	Pulmonary TB only	232 (58.7%)

**Figure 1:**
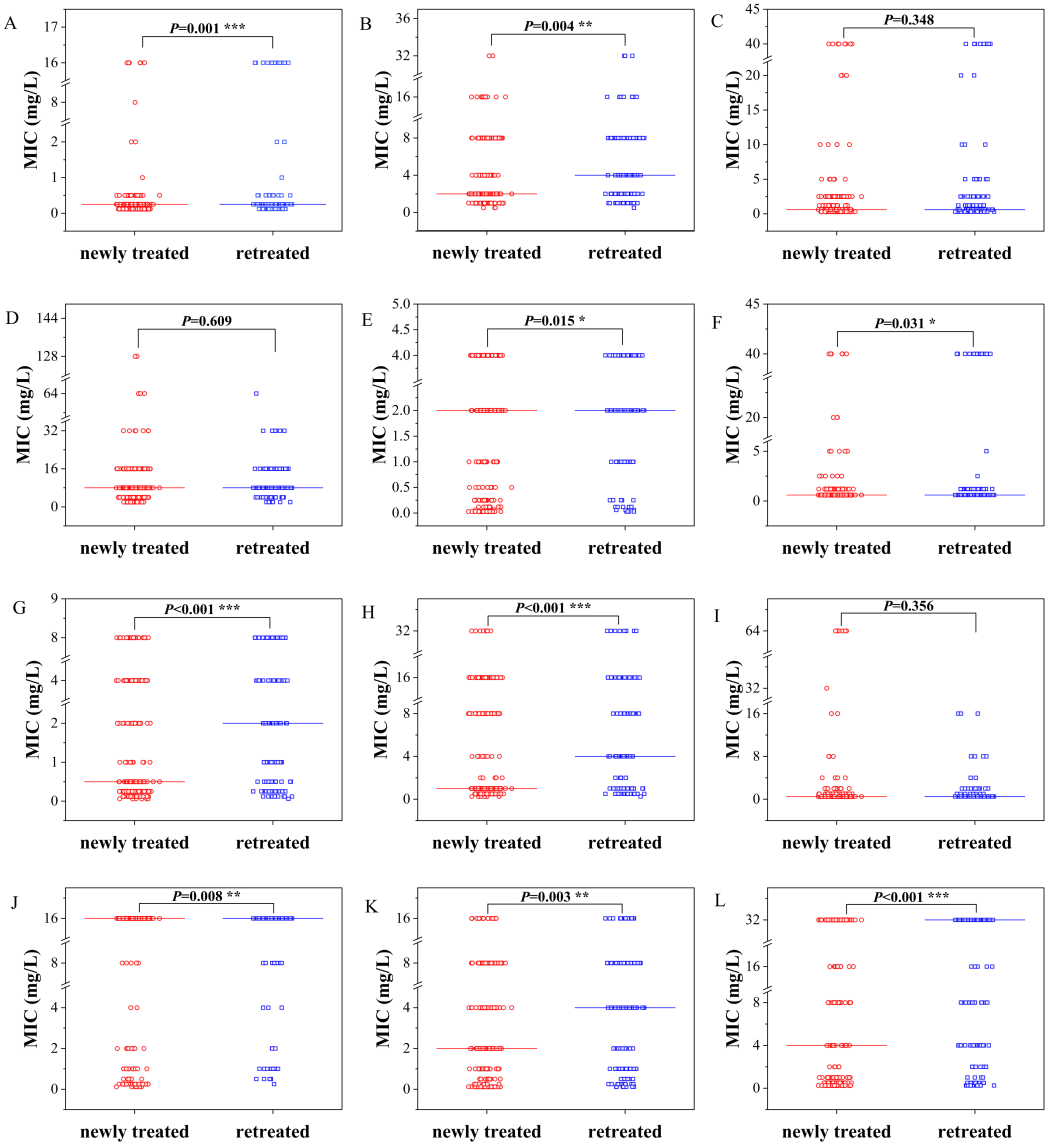
The differences in MIC values of drugs of **(A)** amikacin, **(B)** ethambutol, **(C)** ethionamide, **(D)** cycloserine, **(E)** isoniazid, **(F)** kanamycin, **(G)** moxifloxacin, **(H)** ofloxacin, **(I)** p-aminosalicylic acid, **(J)** rifampicin, **(K)** rifabutin, and **(L)** streptomycin between newly treated and retreated patients with MDR/RR-TB. *P < 0.05, **P < 0.01, ***P < 0.001.

As shown in Figure 1 and Table 2, retreated MDR/RR-TB patients exhibited significantly higher MICs for several drugs compared to newly treated patients, including ethambutol (*p* = 0.004), isoniazid (*p* = 0.015), rifampicin (*p* = 0.008), moxifloxacin (*p* < 0.001), ofloxacin (*p* < 0.001), and streptomycin (*p* < 0.001). No significant differences were observed for ethionamide, cycloserine, or p-aminosalicylic acid (*p* > 0.05). The elevated MICs in retreated patients likely reflect the accumulation of resistance-associated mutations due to prior drug exposure. For fluoroquinolones, mutations in the *gyrA* and *gyrB* genes are known to reduce drug-target binding (Farhat et al., 2017), leading to higher MICs. Similarly, rifampicin resistance typically arises from *rpoB* mutations (Suthum et al., 2020), which were more uniformly present in retreated patients, as evidenced by the narrow MIC distribution (16.0 [16.0–16.0] mg/L). For isoniazid, *katG* and *inhA* promoter mutations confer resistance, with prior treatment potentially selecting for strains carrying these mutations (Liu Y. et al., 2021; Liu C. F. et al., 2021). Conversely, the absence of significant MIC differences for cycloserine and p-aminosalicylic acid suggests that resistance to these drugs develops less readily, possibly due to higher fitness costs or lower selective pressure from prior treatment. These agents may therefore remain effective in retreated patients. Overall, these findings highlight the impact of treatment history on drug resistance profiles and underscore the need for rapid genotypic and phenotypic drug susceptibility testing before retreatment to guide individualized therapy.

**Table 2:** The comparison of MIC values [Median (P_25_, P_75_)] between newly treated and retreated patients with MDR/RR-TB.

Drug	MIC (mg/L)		*p*-value
	Newly treated patients (*n* = 242)	Retreated patients (*n* = 153)	
Amikacin	0.25 (0.12, 0.50)	0.25 (0.12, 0.25)	0.001
Ethambutol	2.0 (2.0, 8.0)	4.0 (2.0, 8.0)	0.004
Ethionamide	0.6 (0.3, 2.5)	0.6 (0.3, 1.2)	0.348
Cycloserine	8.0 (4.0, 16.0)	8.0 (8.0, 16.0)	0.609
Isoniazid	2.0 (0.25, 4.0)	2.0 (2.0, 4.0)	0.015
Kanamycin	0.6 (0.6, 1.2)	0.6 (0.6, 1.2)	0.031
moxifloxacin	0.5 (0.25, 2.5)	2.0 (0.25, 4.0)	<0.001
Ofloxacin	1.0 (1.0, 8.0)	4.0 (1.0,16.0)	<0.001
P-aminosalicylic acid	0.5 (0.5, 1.0)	0.5 (0.5, 0.5)	0.356
Rifampicin	16.0 (2.0,16.0)	16.0 (16.0, 16.0)	0.008
Rifabutin	2.0 (0.5, 8.0)	4.0 (1.0,8.0)	0.003
Streptomycin	4.0 (0.5, 32.0)	32.0 (4.0, 32.0)	<0.001

**Figure 2:**
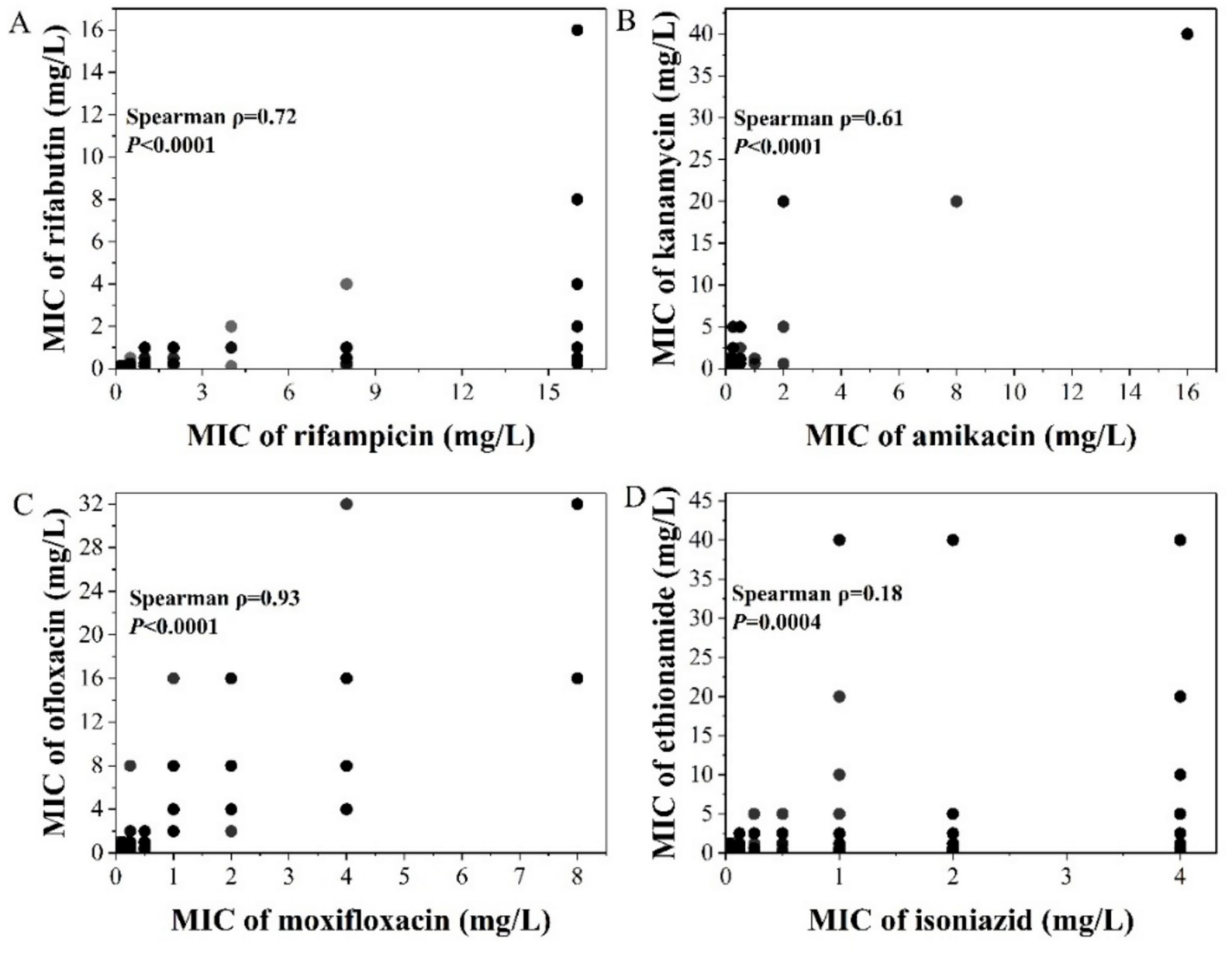
The correlations of MIC values between **(A)** rifampicin and rifabutin, **(B)** amikacin and kanamycin, **(C)** moxifloxacin and ofloxacin, **(D)** isoniazid and ethionamide.

As shown in Figure 2, strong positive correlations were observed between MIC values of moxifloxacin and ofloxacin (*ρ* = 0.93, *p* < 0.0001). Moderate correlations were found between MIC values of rifampicin and rifabutin (*ρ* = 0.72, *p* < 0.0001) and between amikacin and kanamycin (*ρ* = 0.61, *p* < 0.0001), while a weak but significant correlation was noted between isoniazid and ethionamide (*ρ* = 0.18, *p* = 0.0004). The strong correlation between moxifloxacin and ofloxacin reflects cross-resistance driven by *gyrA/B* mutations in the quinolone resistance-determining region (Sun et al., 2024). The moderate rifampicin-rifabutin correlation suggests partial cross-resistance, which may be attributed to some *rpoB* mutations confer high-level rifampicin resistance but spare rifabutin (Schön et al., 2013), supporting its potential use in selected patients. Similarly, the moderate correlation between amikacin and kanamycin indicates cross-resistance, likely due to shared resistance mechanisms involving *rrs* or *eis* mutations (Guo et al., 2025). The weak isoniazid-ethionamide correlation implies limited cross-resistance, as ethionamide resistance primarily involves *ethA* mutations rather than *katG* or *inhA* (Ushtanit et al., 2023), indicating that ethionamide may remain effective in some isoniazid-resistant cases. These findings highlight the importance of individual drug susceptibility testing to guide optimized MDR-TB regimens.

**Figure 3:**
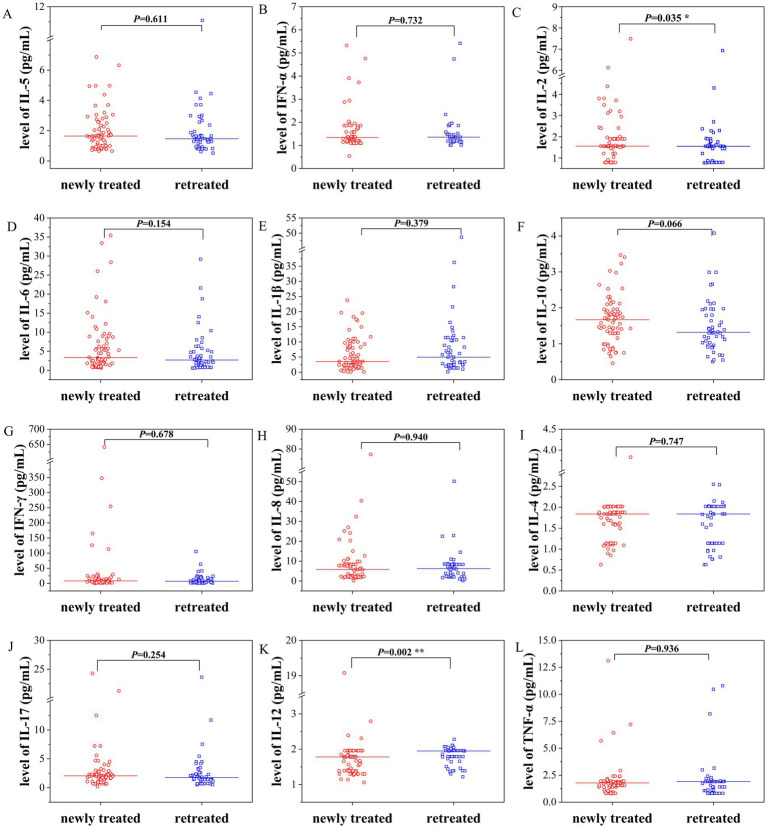
The differences in the levels of plasma inflammatory cytokines of **(A)** IL-5, **(B)** IFN-*α*, **(C)** IL-2, **(D)** IL-6, **(E)** IL-1β, **(F)** IL-10, **(G)** IFN-γ, **(H)** IL-8, **(I)** IL-17, **(J)** IL-4, **(K)** IL-12, and **(L)** TNF-α between newly treated and retreated patients with MDR/RR-TB performed inflammatory factors detection. *P < 0.05, **P < 0.01.

During the clinical diagnosis and treatment process, plasma inflammatory factor testing was performed on 120 out of 395 MDR/RR-TB patients, including 70 newly treated patients and 50 retreated patients. As shown in Figure 3 and Table 3, most plasma inflammatory cytokines showed no significant differences between newly treated and retreated MDR/RR-TB patients. However, statistically significant differences were observed for IL-2 and IL-12. Retreated patients had significantly lower IL-2 levels than newly treated patients (1.55 *vs.* 1.56 pg/mL, *p* = 0.035). IL-2 is primarily secreted by activated T cells and is critical for anti-tuberculosis immunity. As a hypothesis-generating interpretation, the decreased IL-2 levels in retreated patients may reflect T-cell exhaustion due to prolonged or recurrent infection (Shen et al., 2022). Conversely, retreated patients exhibited significantly higher IL-12 levels (1.95 *vs.* 1.78 pg/mL, *p* = 0.002). IL-12 drives Th1 differentiation and IFN-*γ* production, and one possible hypothesis is that its elevation might represent a compensatory immune response to overcome persistent infection in the setting of relative T-cell dysfunction (Kathamuthu et al., 2023). However, given the modest absolute differences between the two groups (IL-2: 0.01 pg/mL; IL-12: 0.17 pg/mL) and the cross-sectional study design, these mechanistic interpretations remain speculative. No significant differences were found for the other ten cytokines assessed (all *p* > 0.05), suggesting that retreatment history has a limited impact on the overall inflammatory milieu. These exploratory findings require validation in future studies.

**Table 3:** The comparison of levels of plasma inflammatory cytokines [Median (P25, P75)] between newly treated and retreated patients with MDR/RR-TB.

Inflammatory factor	Levels of plasma inflammatory cytokines (pg/mL)		*p*-value
	Newly treated patients (*n* = 70)	Retreated patients (*n* = 50)	
IL-5	1.65 (1.00, 2.52)	1.47 (0.93, 2.42)	0.611
IFN-α	1.34 (1.18, 1.72)	1.36 (1.19, 1.49)	0.732
IL-2	1.56 (1.46, 1.91)	1.55 (0.79, 1.76)	0.035
IL-6	3.33 (1.76, 8.36)	2.69 (1.41, 5.37)	0.154
IL-1β	3.53 (2.25, 9.29)	4.96 (2.38, 10.88)	0.379
IL-10	1.67 (1.29, 1.96)	1.32 (0.96, 1.95)	0.066
IFN-γ	8.40 (3.84, 16.00)	7.14 (3.53, 17.38)	0.678
IL-8	5.81 (2.07, 8.35)	6.25 (2.13, 8.34)	0.940
IL-17	2.06 (1.40, 2.60)	1.77 (1.19, 2.59)	0.254
IL-4	1.84 (1.14, 2.01)	1.84 (1.14, 2.02)	0.747
IL-12	1.78 (1.38, 1.96)	1.95 (1.71, 1.96)	0.002
TNF-α	1.79 (1.41, 1.92)	1.91 (1.12, 1.95)	0.936

As shown in Supplementary Table S1, we analyzed the correlation between MIC ranges and treatment outcomes (success *vs.* failure) in MDR/RR-TB patients. Several MIC ranges were nominally associated with treatment failure, including isoniazid (0.12 mg/L < MIC≤1 mg/L, *p* = 0.0010; 1 mg/L < MIC≤4 mg/L, *p* = 0.0014), moxifloxacin (0.5 mg/L < MIC≤4 mg/L, *p* = 0.0016), ofloxacin (2 mg/L < MIC≤8 mg/L, *p* = 0.0019), and streptomycin (MIC≤1 mg/L, *p* = 0.0155; MIC>8 mg/L, *p* = 0.0017). However, these unadjusted comparisons did not account for potential confounding factors. To address the potential influence of other factors, we performed multivariable logistic regression adjusting for age, sex, treatment history, and diabetes (the most frequently observed comorbidity in this cohort), with MIC values log_2_-transformed as continuous variables (Table 4). After adjustment, ethambutol demonstrated a notable association with treatment failure (aOR = 1.620, 95% CI: 1.143–2.295, *p* = 0.007), indicating a 62% increase in the risk of treatment failure for each log_2_-unit increase in ethambutol MIC. This finding suggests that modest elevations in ethambutol MIC may adversely impact treatment outcomes. Similarly, isoniazid also showed a positive association with treatment failure (aOR = 1.315, 95% CI: 1.048–1.651, *p* = 0.018), corresponding to a 31.5% increase in failure risk per log_2_-unit increase in MIC. Furthermore, after multiple comparisons across 12 drugs, the FDR-adjusted q-values for ethambutol and isoniazid were 0.084 and 0.108, respectively (both>0.05), and for all other drugs exceeded 0.10, indicating none of the associations remained statistically significant. And the previously observed associations for moxifloxacin, ofloxacin, and streptomycin were no longer significant after adjusting for age, sex, treatment history, and diabetes, suggesting that those unadjusted findings may have been confounded by these baseline characteristics. Unfortunately, due to unavailability of the data of other clinically important factors including resistance category, disease severity, regimen composition, and adherence, our multivariable models did not adjust for these variables, which should be explicitly acknowledged as a limitation. Given the modest absolute differences observed and the exploratory nature of these analyses, our findings should be interpreted as hypothesis-generating rather than inferential. These findings show some deviation from those of previous study (Tang et al., 2022), which may be due to the limited number of failure events (*n* = 35) and adjustment for confounding factors. Therefore, whether MIC levels provide independent prognostic value for treatment outcomes in MDR/RR-TB patients remains to be established in larger, well-powered cohorts. In conclusion, while our exploratory analysis suggests potential associations between ethambutol or isoniazid MIC values and treatment failure, these findings require further validation before any prognostic claims can be made.

**Table 4:** Multivariable logistic regression analysis of the association between MIC levels and treatment outcomes in patients with MDR/RR-TB.

Drug	Adjusted odds ratio (aOR)	95% confidence interval (CI)	*p*-value
Amikacin	0.975	0.802–1.186	0.803
Ethambutol	1.620	1.143–2.295	0.007
Ethionamide	0.837	0.666–1.050	0.125
Cycloserine	1.168	0.742–1.837	0.503
Isoniazid	1.315	1.048–1.651	0.018
Kanamycin	1.033	0.828–1.290	0.772
Moxifloxacin	0.952	0.778–1.164	0.632
Ofloxacin	0.907	0.732–1.124	0.373
P-aminosalicylic acid	1.119	0.679–1.843	0.659
Rifampicin	1.154	0.916–1.454	0.223
Rifabutin	1.145	0.944–1.390	0.170
Streptomycin	0.955	0.801–1.137	0.604

## 4 Conclusion

In summary, this study of 395 MDR/RR-TB patients demonstrates that the cohort exhibits a high proportion of pre-XDR-TB (48.4%) and a suboptimal treatment success rate (66.8%). Retreated patients show significantly elevated MICs for multiple drugs and distinct immune dysregulation characterized by decreased IL-2 and increased IL-12 levels. Strong cross-resistance exists between moxifloxacin and ofloxacin, while moderate partial cross-resistance were found between rifampicin and rifabutin and between amikacin and kanamycin. Elevated MICs for ethambutol and isoniazid were associated with treatment failure after multivariable adjustment. However, these associations did not retain statistical significance after Benjamini-Hochberg FDR correction for multiple comparisons. Therefore, these findings should be considered exploratory and hypothesis-generating rather than conclusive evidence of independent prognostic value for quantitative MIC testing. Given the limited sample size, and the lack of adjustment for several clinically important factors including resistance category, disease severity, regimen composition, and adherence, these findings require validation in larger cohorts. If confirmed in future studies, they would suggest a potential role for considering MIC results in individualized regimen design, along with comprehensive patient support to reduce loss to follow-up in MDR/RR-TB.

## Data Availability

The original contributions presented in the study are included in the article/Supplementary material, further inquiries can be directed to the corresponding authors.
