# Supplementary material for: Clinical characteristics, drug resistance profiles, and inflammatory cytokine signatures in newly treated versus retreated MDR/RR-TB patients: associations with treatment outcomes
Source: Front Microbiol. 2026 Jul 1;17:1871422. doi: 10.3389/fmicb.2026.1871422 (PMC13368717; doi:10.3389/fmicb.2026.1871422)
Supplement: Supplementary file 1 [file Table_1.DOCX]

**Supplementary Information**

**Clinical characteristics, drug resistance profiles, and inflammatory cytokine signatures in newly treated versus retreated MDR/RR-TB patients: Associations with treatment outcomes**

Yiyan Song^1, *^, Dawei Yu^1^, Junchi Xu^1^, Yunxia Zhai^1^, Huafeng Song^1^, Hui Chen^1^, Jianping Zhang^2, *^, Ping Xu^1, *^, Fanghua Li^1, *^

^1^ Department of Clinical Laboratory, The Fifth People’s Hospital of Suzhou, The Affiliated Infectious Diseases Hospital of Soochow University, Suzhou, 215000, China

^2^ Department of Tuberculosis, The Fifth People’s Hospital of Suzhou, The Affiliated Infectious Diseases Hospital of Soochow University, 215000, China

*Corresponding Author: Yiyan Song, songyiyan94@163.com; Jianping Zhang, zhangjianping_yb@suda.edu.cn; Ping Xu, 573311485@qq.com; Fanghua Li, lifanghua_2019@qq.com.

**Table S1.** The correlations of MIC ranges between the treatment outcomes of success and failure in patients with MDR/RR-TB.

| Drug | MIC range  (mg/L) | Treatment outcome | | *P* value |
| --- | --- | --- | --- | --- |
|  |  | Success (n=221) | Failure (n=35) |  |
| Amikacin | ≤0.12 | 75 | 10 | 0.5312 |
|  | 0.12＜MIC≤4 | 133 | 21 | 0.9838 |
|  | ＞4 | 13 | 4 | 0.2208 |
| Ethambutol | ≤1 | 36 | 10 | 0.0860 |
|  | 1＜MIC≤4 | 114 | 16 | 0.5187 |
|  | 4＜MIC≤16 | 68 | 8 | 0.3411 |
|  | ＞16 | 3 | 1 | 0.5063 |
| Ethionamide | ≤2.5 | 198 | 33 | 0.3848 |
|  | 2.5＜MIC≤20 | 12 | 2 | 0.9452 |
|  | ＞20 | 11 | 0 | 0.1773 |
| Cycloserine | ≤4 | 53 | 10 | 0.5581 |
|  | 4＜MIC≤16 | 157 | 24 | 0.7655 |
|  | ＞16 | 11 | 1 | 0.5490 |
| Isoniazid | ≤0.12 | 25 | 4 | 0.9839 |
|  | 0.12＜MIC≤1 | 24 | 11 | 0.0010 |
|  | 1＜MIC≤4 | 150 | 14 | 0.0014 |
|  | ＞4 | 22 | 6 | 0.2055 |
| Kanamycin | ≤0.6 | 147 | 25 | 0.5652 |
|  | 0.6＜MIC≤5 | 59 | 6 | 0.2276 |
|  | ＞5 | 15 | 4 | 0.3304 |
| Moxifloxacin | ≤0.5 | 107 | 11 | 0.0610 |
|  | 0.5＜MIC≤4 | 77 | 22 | 0.0016 |
|  | ≥8 | 37 | 2 | 0.0916 |
| Ofloxacin | ≤2 | 111 | 11 | 0.0386 |
|  | 2＜MIC≤8 | 52 | 17 | 0.0019 |
|  | ＞8 | 59 | 7 | 0.4000 |
| P-aminosalicylic acid | ≤0.5 | 171 | 28 | 0.7288 |
|  | 0.5＜MIC≤2 | 40 | 7 | 0.7873 |
|  | ＞2 | 10 | 0 | 0.1992 |
| Rifampicin | ≤1 | 36 | 6 | 0.8992 |
|  | 1＜MIC≤8 | 11 | 4 | 0.1311 |
|  | 8＜MIC≤16 | 38 | 3 | 0.1962 |
|  | ＞16 | 136 | 22 | 0.8815 |
| Rifabutin | ≤0.5 | 47 | 7 | 0.8645 |
|  | 0.5＜MIC≤4 | 94 | 16 | 0.7240 |
|  | ＞4 | 80 | 12 | 0.8265 |
| Streptomycin | ≤1 | 77 | 5 | 0.0155 |
|  | 1＜MIC≤8 | 35 | 6 | 0.8448 |
|  | ＞8 | 89 | 24 | 0.0017 |
